# Arterial and venous flow dynamics are modified by age in the non-human primate

**DOI:** 10.1162/IMAG.a.66

**Published:** 2025-07-07

**Authors:** Germain Arribarat, Muriel Mescam, Franck Desmoulin, Caroline Fonta

**Affiliations:** Univ Toulouse, CNRS, CerCo, Toulouse, France; Univ Toulouse, Inserm, ToNIC, Toulouse, France

**Keywords:** cerebral blood flow, brain fluids, craniospinal system, haemodynamics, intracranial vessels, marmoset (*Callithrix jacchus*)

## Abstract

Growing evidence suggests that vascular abnormalities contribute to neurodegenerative processes during ageing. With the anticipated rise in the elderly population, we aimed to characterise arterial and venous flow dynamics, which reflect cardio-cerebro-vascular conditions, using a preclinical model: the marmoset monkey. We employed MRI protocols to image brain and intracranial vessel anatomy. We adapted the phase-contrast MRI protocol to measure blood dynamics synchronised with the ECG signal in the carotid arteries, basilar trunk, superior sagittal, and straight sinuses. We imaged both young and old healthy marmosets. Our analysis revealed a similar chronological sequence of maximal blood flow values in the various arteries and sinuses for both young and old marmosets; however, the peaks occurred earlier in the cardiac cycle for the older group. Furthermore, we observed shorter arterio-venous delays in older marmosets, suggesting ageing-related cardiovascular and cerebrovascular changes. We analysed the temporal progression of arterial and venous blood volumes within the cranial system at the cardiac cycle scale, providing valuable insights into the constraints on the craniospinal system. An illustration of the relationships between input flow and output flow revealed a characteristic hysteresis effect, indicating an age-dependent behaviour of the venous system.

## 1 Introduction

Brain activity, which includes processes such as receiving, organising, and transmitting information, is supported by systems that require large amounts of energy. This constraint creates a challenge because the brain has a limited capacity for energy storage. As a result, maintaining regular brain activity is critically dependent on the cardiovascular system to deliver nutrients and oxygen efficiently. However, ageing deteriorates cardiovascular functionality, leading to an increased risk of cerebrovascular diseases. As accumulating evidence supports the hypothesis that vascular defects can initiate and contribute to neurodegenerative mechanisms (De La Torre, 2004; Iadecola, 2013; Rocha et al., 2022; Zlokovic, 2005), the links between anatomo-functional changes of the cardiovascular–cerebral system and alteration or loss of cerebral activity and cognitive abilities during ageing are being intensively analysed and deserve further exploration due to the projected increase of the older adult population over the following decades (UN.org, 2023).

Being anatomically and phylogenetically closer to humans than the commonly used rodent species, the common marmoset is an invaluable non-human primate (NHP) model for studying human health and diseases. Marmosets have a shorter lifespan than many other NHPs. They are usually considered aged by 8 years. They survive as long as 10 years in the wild and longer in captivity (Abbott et al., 2003; Ross & Salmon, 2019; Tardif et al., 2011; Ward et al., 2014). Recent studies demonstrate cognitive impairment in aged marmosets (Rothwell et al., 2022; Sadoun et al., 2019), positioning this NHP as a suitable model for preclinical ageing research. However, signs of brain and cardiovascular system ageing in this species are similar to those reported in humans; for example, several studies have reported beta-amyloid deposits (Geula et al., 2002), loss of calbindin in basal forebrain cholinergic neurons (Wu et al., 2003), accumulation of synaptic alpha-synuclein protein (Kobayashi et al., 2016), Tau hyperphosphorylation (Rodriguez-Callejas et al., 2016), and decreased neurogenesis (Leuner et al., 2007).

Regarding the heart, reduced ventricular volume (possibly due to remodelling of the myocardium (Moussavi et al., 2020), which produces diffuse interstitial fibrosis (Khan et al., 2021), has been reported in marmoset monkeys older than 10 years. In young adult marmosets aged between 1 and 2.5 years, inflammatory cell infiltration, perivasculitis, mineralisation, and focal myocarditis were common but not accompanied by clinical findings (Chamanza et al., 2006). Age alters cardiovascular health in the marmoset by increasing mean arterial pressure (MAP) and diastolic but not systolic pressure (Mietsch et al., 2016; Ross & Salmon, 2019). Literature on vessel ageing in marmosets is scarce: histochemical changes in the microvasculature have been described (Sobin et al., 1992), and atherosclerotic lesions have been observed in macrovessels (Ross & Salmon, 2019; Wachtman et al., 2011). However, no study has quantitatively examined the modalities of arterial inflow and venous outflow in the marmoset brain, which, respectively, reflect the state of the cardiovascular system and the condition of the cerebral vascular bed.

Therefore, we evaluated the feasibility of non-invasively indexing vascular functionality to provide benchmark data on the cardio-cerebral vascular axis in healthy phenotypes in young and old adult marmosets. We used MRI to image the anatomy of the brain and intracranial vessels and to measure blood velocity in intracerebral arteries and sinuses throughout the cardiac cycle. We thus calculated different arterial and venous blood flow parameters. From these data, we inferred global brain perfusion and temporal changes in arterial and venous blood volumes inside the cerebral tissue at the scale of the cardiac cycle. Our original results point to significant haemodynamic changes in aged marmosets, at both the cardiovascular and cerebrovascular levels, strengthening the value of this NHP model in cerebrovascular biomedical investigations.

## 2 Material and Methods

### 2.1 Marmoset monkeys

Nineteen monkeys (*Callithrix jacchus*) from the marmoset colony housed in the Brain and Cognition Research Center (CerCo, Toulouse, France) were divided into 2 groups: 10 young adults (YA) (6 males and 4 females, with ages ranging from 3.2 to 5 years (median 3.9 years) and with weights ranging from 330 to 430 g (median 375 g), and 9 old adults (OA) (5 males and 4 females, with ages ranging from 8.4 to 9.2 years (median 8.9 years) and weights ranging from 330 to 450 g (median 400 g) (Supplementary Table S1).

All experiments were carried out according to the National Committee for Ethical Reflection on Animal Testing (authorisation number: 05215.03 issued by Ministère de l’Education Nationale, de l’Enseignement Supérieur et de la Recherche).

Data acquisition for the two experimental groups was carried out randomly. We inspected brain anatomy using T2-weighted (T2-W) images to include the animals. Specifically, we ensured the absence of substantial cortical atrophy and examined the location, symmetry, and volume of ventricular structures. Image examination enabled us to assess the symmetry and volume of the hippocampi. Additionally, we confirmed the unobstructed nature of the pericerebral spaces, assessed the condition of the cisterns, and checked for anomalies along the midline, the pituitary location, and the cervico-occipital junction. No signal abnormalities were detected either above or below the cerebellar tentorium. Vessel integrity was checked on time-of-flight (TOF) images reconstructed in different spatial planes.

Marmoset biological data (age, weight, heart rate, sex) and brain anatomical MRI data (T2-W, TOF) were obtained for all 19 marmosets (10 YA and 9 OA). However, two marmosets (one YA and one OA) were excluded due to poor PC-MR image quality. Additionally, venous blood velocity could not be acquired for one OA, and arterial blood velocity data were incomplete for two YA. Consequently, independent analyses of arterial and venous flow included seven YA and eight OA for arterial flow and nine YA and seven OA for venous flow. The paired arterio-venous blood flow coupling analysis was conducted on seven YA and seven OA.

### 2.2 MRI methods

The marmosets were fasted for 4 h and water-deprived for 2 h before MRI scanning. The monkeys were first anaesthetised with an intramuscular injection of 0.2 ml Alfaxan (Alphaxalone, 10 mg/ml) to transfer them from their colony housing cage to the MRI scanner facility. They were then placed in a prone position in a dedicated MR-compatible imaging cell (Minerve équipement vétérinaire, Esternay, France) under oxygen ventilation with 1.5 to 2.5% isoflurane delivered via a nose cone. Their heads were held in place by two ear bars. The imaging cell was continuously heated to 37°C. Two ECG electrodes (Red Dot^TM^ disposable neonatal monitoring electrode, 3 M Health Care, Germany) were positioned on the left hindlimb and right forepaw. A small pneumatic pillow was placed under the marmoset’s chest to detect respiratory rate. The cardiac rate, respiratory rate, and temperature were monitored during experiments (Minerve Physiogard II software). The breath rate was monitored to adjust the anaesthesia concentration if necessary during the MRI acquisitions.

All MRI experiments were conducted using a Bruker BioSpec small animal MRI scanner system (Bruker Biospin MRI GmbH; Ettlingen, Germany) equipped with a 7T (70/16 USR) horizontal bore magnet. For signal detection, a 72 mm transmit quadrature birdcage resonator was used for radio-frequency transmission and a single-loop 30-mm surface coil was positioned on top of the skull for signal reception. The shape of the coil naturally conforms to the spheroidal skull of the marmoset and is self-centering on the skull. The coil was further secured to the animal’s head with a strap, ensuring that its surface remained parallel to the ear bars. The dimensions of the surface coil (30 mm external diameter, 25 mm internal diameter) and the positioning of the targeted arteries and sinuses (20 mm below the coil) ensured an adequate signal-to-noise ratio (SNR) at the base of the brain. The Bruker ParaVision 6.0.1 software was used to acquire the MR data.

**Fig. 1.**
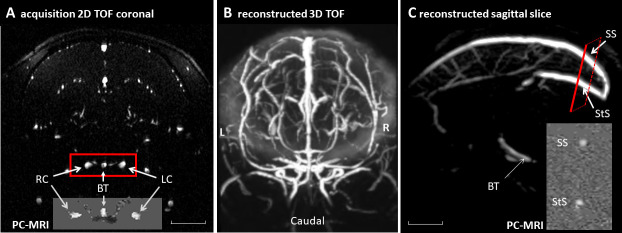
TOF and PC-MRI acquisitions in marmoset monkey brain. TOF images were used to select two scanning planes perpendicular to the flow direction for PC-MRI acquisitions (insets in A and C). (A) A TOF image acquired in the coronal plane was selected to determine the orientation of the slice used for measuring blood velocity in arteries by PC-MRI (inset in A), simultaneously capturing the basilar trunk artery (BT), the left internal carotid artery (LC), and the right internal carotid artery (RC). (B) A 3D reconstruction of TOF images. (C) The sagittal plane, determined from the 3D reconstruction of TOF images, was used to position an oblique slice, perpendicular to the sinuses, for measuring blood velocity in the sinuses by PC-MRI (inset in C), simultaneously capturing the superior sagittal sinus (SS) and the straight sinus (StS). Scale bar = 5 mm. L: left, R: right.

The measurements were obtained in the morning (10:00–12:00). MRI acquisitions took between 90 and 105 min per marmoset to acquire T2-W, time of flight (TOF), and velocity images (PC-MRI) sequentially. T2-W images were acquired in the horizontal plane with the following parameters: TR/TE: 6859/36 ms; FOV: 48 x 48 mm; matrix = 240 x 240; pixels = 200 x 200 µm; 60 slices of 400 µm; averages: 4; acquisition time: 13 min. Intracranial vessels were visualised by 2D magnetic resonance angiography time-of-flight (TOF). Images were acquired in the coronal plane with the following parameters: TR/TE = 11/3 ms; flip angle = 80°; FOV: 48 x 48 mm; matrix = 320 x 320; voxels = 150 x 150 µm; 140 slices of 300 µm; averages: 4; acquisition time: 26 min. The TOF images and 3D reconstruction were used to orient the two scanning planes perpendicular to the flow direction in the arteries and sinuses of interest in the PC-MRI study (Fig. 1). Further details on the acquisition procedure are provided in the Supplementary Figure S1.

Blood flow images were acquired using a two-dimensional cine PC-MRI pulse sequence with prospective cardiac gating. An intracranial coronal section across the inferior part of the Circle of Willis was selected to simultaneously measure blood velocity in both the left (LC) and right (RC) internal carotids and the terminal part of the basilar artery trunk (BT) (before it bifurcates into the two posterior cerebral arteries) (Fig. 1A). The slice for acquiring blood velocity in the sinuses was positioned based on a reconstructed sagittal TOF image and it was placed upstream of the division of the straight sinus (StS) into two branches, at a point of low curvature of the upper sagittal sinus (SS) (Fig. 1C). PC-MRIs were sequentially acquired for arterial and venous blood. Care was taken to set the acquisition plane as perpendiculary as possible to the expected flow direction in the vessels and sinuses to prevent underestimation or a lack of variation in flow velocity (Tang et al., 1993).

**Fig. 2.**
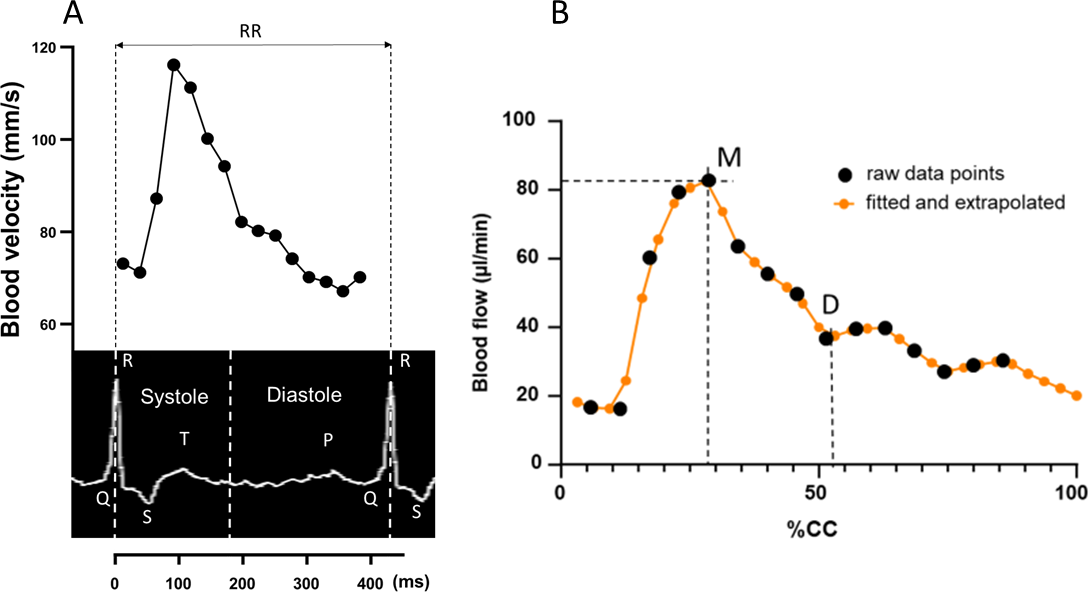
Blood velocity acquisition and blood flow time course as a function of cardiac activity. (A) Representative velocity curve acquired from a carotid. PC-MRI acquisition (top) was synchronised with the ECG signal R-peaks (bottom). Velocity was acquired at regular intervals (every 25 ms) over 80% of the cardiac cycle. The blood velocity profile thus corresponds to the sequence of the whole ventricular systole and a large part of the ventricular diastole. (B) Blood flow was calculated as the product of blood velocity and vessel cross-sectional area obtained from PC-MRIs (black dots). To normalise the different acquisitions across all marmosets, blood flow values were linearly interpolated and extrapolated (orange dots) over the entire cardiac cycle (CC) with 32 steps (one step = 3.125% CC). The maximum blood flow value (M) was specified along with the corresponding % CC. This was determined for all arterial and venous blood flow profiles. The dicrotic notch (D) is commonly used to indicate the break between the steep and slow decrease in the arterial flow, marking the systole end.

Because eddy currents are generated in the ECG acquisition wires, acquisition started at the onset of the R-wave and ended before the subsequent R-wave to ensure accurate detection of the cardiac cycle. Thus, 12–22 frames were obtained per cardiac cycle (CC) as a function of heart rate (Fig. 2A). On average, 75% of the cardiac cycle was covered (79.2 ± 5.8% in young marmosets; 69.8 ± 15.9% in old marmosets) (Supplementary Table S1). The MRI parameters were as follows: TR/TE = 15 /4.2 ms; flip angle = 20°; FOV: 30 x 24 mm; matrix = 200 x 160; voxel size: 150 x 150 x 1000 µm; acquisition time: 26 min. A one-sided velocity encoding (VENC) was set at 50 cm/s for the arteries and 20 cm/s for the sinuses. No aliasing was observed, which would have resulted from a VENC that was too small.

### 2.3 Data analysis

#### 2.3.1 Tissue segmentation

An image processing pipeline was applied to the T2-W images. All T2-W marmoset brain images were initially aligned to the anterior-posterior commissural (AC-PC) axis. These images were then normalised for intensity and segmented into probability maps for grey matter (GM), white matter (WM), and cerebrospinal fluid (CSF). The segmentation was performed using the SPM software (Friston et al., 1994), incorporating the marmoset MRI Standard Brain probabilistic tissue templates (Gennai et al., 2015).

#### 2.3.2 Vessel segmentation (TOF, PC-MRI)

To analyse PC images, we utilised an in-house imaging analysis software (SISYPHE, Sisyphe Software for Neuroimaging; Tensaouti et al., 2008), especially for delineating the vessel lumen. The image analyses were conducted blindly. Vessel segmentation within PC-MRIs involved defining regions of interest (ROIs) using both automated and manual methods. For the arterial section, boundaries were set around BT, RC, and LC. In the venous portion, both SS and StS were delineated.

We set a minimum segmentation threshold to measure the vessel area at twice the image background noise standard deviation (Supplementary Fig. S1). This threshold, grounded on the signal-to-noise ratio, provided precise and robust segmentation of the ROI, ensuring a clear distinction between the desired signal and background noise. Considering the potential ROI variations among the images captured throughout the cardiac cycle, we applied this signal-to-noise ratio-based thresholding to each image. Addressing these ROI variations was crucial for accurately depicting blood flow velocity, as it allowed us to account for the effects of blood pulsation on both the position of the vessel lumen within the slice and its cross-sectional area.

#### 2.3.3 Blood velocity and blood flow calculation (PC-MRI)

On average, the cross-section at the vessel lumen obtained for all measurements (n = 112 to 130 measures per vessel/sinus and per age group) covered (22 ± 10 and 20 ± 7), (23 ± 13 and 25 ± 7), (31 ± 9 and 28 ± 14), and (14 ± 5 and 23 ± 8), and (13 ± 5 and 22 ± 5) voxels for RC, LC, BT, StS, and SS in old and young marmosets, respectively. Blood flow is calculated as the product of the blood velocity (cm/s) (as defined above) multiplied by the cross-sectional area of the vessel/sinus lumen (mm^2^). These spatial resolution conditions allow for accurate determination of blood flow by PC MRI (P. Liu et al., 2023; Tang et al., 1993), defined from the mean of the velocities measured in each of the voxels within the vessel section, for each acquisition frame.

To assess measurement repeatability, we conducted two independent analyses on a subset of data (n = 4 marmosets: 2 young adults, 2 old adults). Each dataset was segmented and processed twice in a blinded manner, using the same pipeline described above (including registration, ROI delineation, and computation of velocity and cross-sectional area). We then calculated the coefficient of variation (CV) for vessel cross-sectional areas and their mean velocities, and the intra-class correlation coefficient (ICC) for blood flow. These analyses demonstrated the high reproducibility of our method, despite the expected propagation of error from area and velocity estimates. Detailed repeatability metrics are provided in Supplementary Table S2.

#### 2.3.4 Data normalisation

The blood flow could not be calculated for the entire duration of the cardiac cycle (cf. 2.2 above) since, on average, acquisitions covered 75% of the cardiac cycle, with an acquisition step every 2.7 to 6.9% of the cardiac cycle (CC). These acquisition conditions were not significantly different between the two groups of age (data not shown). In order to do compatible analyses between the arterial and the venous compartments, as well as inter-individual comparisons, we first extrapolated blood flow data (using linear extrapolation over the entire CC (100% CC), and then linearly interpolated the data to provide blood flow values at regular steps of the CC. This approach ensured consistent data across all marmosets, vessels, and sinuses. Therefore, the blood flow data were distributed into 32 steps of the CC, each representing 3.125% CC (Fig. 2B; Supplementary Table S3).

The maximum value of blood flow, along with the corresponding % CC, was determined on flow profiles (point M, Fig. 2B). The dicrotic notch could also be ascribed to the arterial blood flow profile (point D), corresponding to the break in slope at the systole end (Fig. 2B) (Loh et al., 2022).

### 2.4 Statistical analysis

Comparisons were performed, unless otherwise specified, using bivariate analysis with nonparametric tests (Mann–Whitney test, paired-Wilcoxon test). Associations between sex and age groups were tested using a Fisher’s exact test. The level of statistical significance (p-value) was set at 0.05, with Bonferroni correction applied in case of multiple comparisons. Values are expressed as mean ± standard deviation.

## 3 Results

### 3.1 Marmoset population

The two age groups of marmosets were characterised based on biological and anatomical grounds. Sex representation between the groups is comparable (Fisher test, p = 0.46). The body weight averages 382 ± 40 g in the whole population. No significant difference was found between young and old marmosets (Table 1) or between male and female marmosets with weights of 377 ± 43 g (n = 11) and 387 ± 37 g (n = 8), respectively (p = 0.6, t-test).

**Table 1.** Biological, MRI anatomical and blood flows data in young (YA) and old (OA) marmosets.

	YA (n)	OA (n)	p
**Age** (years)	3.9 ± 0.6 (10)	8.8 ± 0.3 (9)	<0.0001
**n** (male/female)	10 (6/4)	9 (5/4)	0.460
**Weight** (g)	373 ± 41 (10)	391 ± 38 (9)	0.361
**Heart rate** (bpm)	189 ± 58 (9)	152 ± 42 (8)	0.147
**Brain volume** (µl)	7695 ± 803 (10)	8206 ± 628 (9)	0.133
**Grey matter volume** (µl)	4084 ± 628 (10)	4256 ± 466 (9)	0.720
**White matter volume** (µl)	2669 ± 260 (10)	2831 ± 292 (9)	0.211
**CSF volume** (µl)	941 ± 335 (10)	1119 ± 337 (9)	0.457
**Arterial blood volume**			
**Basilar trunk artery**			
µl/ cardiac cycle	19.0 ± 4.3 (7)	30.3 ± 6.7 (7)	0.004
% arterial input	40.3 ± 6.2 (7)	45.2 ± 10.2 (7)	0.298
**Right carotid**			
µl/ cardiac cycle	13.2 ± 5.1 (7)	19.3 ± 6.7 (7)	0.097
% arterial inputs	27.2 ± 6.9 (7)	27.7 ± 5.1 (7)	0.871
**Left carotid**			
µl/ cardiac cycle	15.5 ± 4.5 (7)	19.5 ± 9.0 (7)	0.318
% arterial inputs	32.5 ± 6.9 (7)	27.1 ± 9.0 (7)	0.230
**Total carotids**			
µl/ cardiac cycle	28.7 ± 7.8 (7)	38.8 ± 14.6 (7)	0.128
% arterial inputs	59.7 ± 6.2 (7)	54.8 ± 10.2 (7)	0.298
**Total arteries**			
µl/ cardiac cycle	47.7 ± 10.9 (7)	69.2 ± 18.4 (7)	0.026
**Perfusion** (all arteries)ml/ min	9.0 ± 2.3 (7)	11.0 ± 4.3 (7)	0.383
**CBF**ml/min/100 g tissue	126.4 ± 20.8 (7)	148.8 ± 58.6 (7)	0.535
**Venous blood volume**			
**SS**			
µl/ cardiac cycle	6.2 ± 3.7 (7)	5.6 ± 2.4 (7)	0.901
% venous blood volume	54.6 ± 17.3 (7)	48.6 ± 13.8 (7)	0.620
**StS**			
µl/ cardiac cycle	6.8 ± 1.8 (7)	5.7 ± 3.2 (7)	0.456
% venous blood volume	45.4 ± 17.3 (7)	51.4 ±13.8 (7)	0.620
**Total sinuses**			
µl/ cardiac cycle	13.0 ± 3.4 (7)	11.3 ± 5.0 (7)	0.535
**Drainage (all sinuses)**ml/min	40.5 ± 11.9 (7)	27.6 ± 14.5 (7)	0.053

Heart rate was averaged on the arterial sequences (nine sequences in the YA group, eight sequences in the OA group; Supplementary Table S1).

The average heart rate (HR) for anaesthetised marmosets was 172 ± 53 bpm (n = 17; Supplementary Table S1) at the start of the PC-MRI experiment. Heart rate did not fluctuate significantly during acquisitions but tended to decline gradually from the start of the arterial to the end of the venous blood flow velocity acquisition. Despite the extended duration of the experiment (90 to 105 min), no significant change was observed, with an HR of 167 ± 50 bpm (Supplementary Table S1) recorded at the end of the experiment (p = 0.77, t-test).

Mean HRs are comparable between young (189 ± 58 bpm) and old marmosets (152 ± 42 bpm) (p = 0.147) (Table 1).

Regarding total brain volume, as well as grey matter (GM) and white matter (WM) volumes, no significant differences were found between age groups (Table 1) or between sexes (p = 0.982 t-test n = 11 males and 8 females). Cerebrospinal fluid (CSF) volume, which corresponds to approximately 1000 µl, occupies 13% of the intracerebral volume, as in humans.

Blood volume supplying or draining the brain during the cardiac cycle (CC) is calculated as the sum of elementary blood volumes delivered during each step of the CC (3.125% CC). These elementary blood volumes result from the integration of blood flow value over the duration (in ms) of the CC step for each marmoset, as cardiac frequency varies from one marmoset to another. Perfusion values normalise the blood input per minute. Cerebral blood flow (CBF) takes into account the individual weight of brain tissue (sum of grey and white matter volumes) multiplied by 1.05 g/ml, the mean value of brain tissue density in marmosets (Stephan et al., 1981). Data are presented as mean ± standard deviation.

### 3.2 Intra-cerebral blood flow dynamics

#### 3.2.1 Chronological sequence of maximal arterial and venous flows

**Fig. 3.**
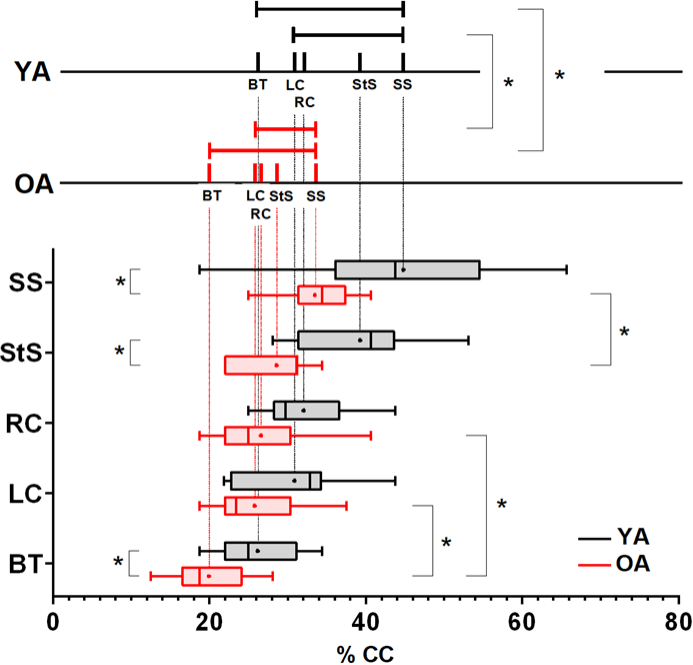
Comparative arrival times of the blood flow peak (M) in the different arteries and sinuses. Lower part, right: comparison between the different inflow or outflow timings in either YA or OA. In YA, no significant difference can be found in the arrival times of the maximal flow, either between BT, RC, and LC or between StS and SS. In contrast, in OA, BT maximal flow input significantly precedes the RC input (p = 0.017, n = 7) and the LC input (p = 0.017, n = 7), and the StS maximal outflow is earlier than the SS one (p = 0.040, n = 7) (non-parametric paired-test with Bonferroni correction for multiple comparisons). Lower part, left: The maximal value of blood flow in OA precedes that in YA in the arterial (BT) as in the venous system (StS, SS). Mann–Whitney tests (p = 0.038 for BT, 0.043 for SS, 0.018 for StS). Upper part: Comparison of arterial input and venous output time course between YA and OA. The time intervals are calculated as the means of the difference in arrival times (expressed in %CC) of maximal blood flow in the different vessels or sinuses. During the cardiac cycle, the time interval between the arterial (BT or LC) and the venous (SS) maximal blood flow is significantly smaller in old than in young adult marmosets (p = 0.030 BT/SS, p = 0.013 LC/SS). CC: cardiac cycle, BT: basilar trunk artery, RC: right carotid, LC: left carotid, StS: straight sinus, SS: superior sagittal sinus. YA: young adult marmoset group, OA: old adult marmoset group. Data are presented as boxplots with whiskers for min and max values (a black dot indicates the mean). * Indicates a statistically significant difference.

**Fig. 4.**
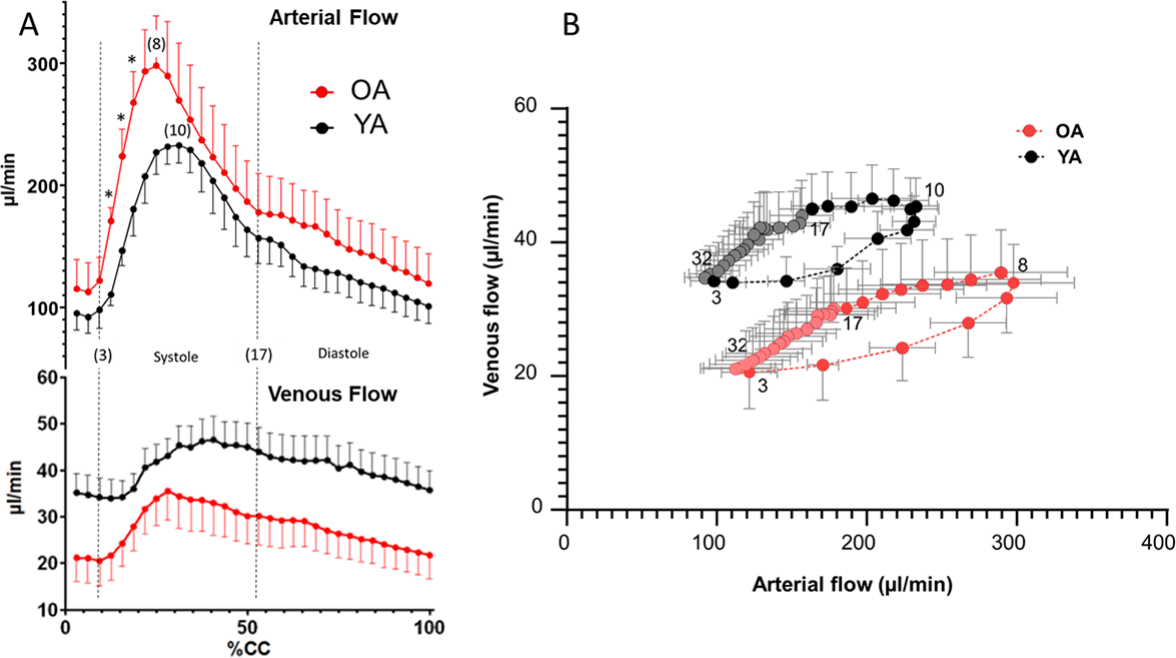
Time course of arterial and venous blood flows along the cardiac cycle (CC). (A) Arterial flow is the sum of the blood flows measured in the basilar trunk artery and the two carotids. The venous flow is the sum of blood flow measured in the straight and superior sagittal sinus. The maximal value of arterial blood flow occurs at different steps of the CC: at step 10 for young (YA) and at step 8 for old (OA) marmosets. Step 3 represents the start of the large arterial inflow to the brain, and step 17 marks the dicrotic notch, which defines the transition between the systole and diastole cardiac phases. Data are presented as mean ± SEM; n = 7 per age group. Each marmoset provided both arterial and venous flow data. Error bars are shown on one side to improve legibility. *Indicates a significant difference in the flow values (p < 0.05). (B) The relationship between arterial and venous blood flows was established with 32 measurement steps over the CC (synchronised with the R-wave of the EEG, point 1) in young and old adult marmosets. This relationship exhibits a hysteresis phenomenon consisting of three successive periods. The first period involves nonlinear growth, with an increase in arterial flow paired with an out-of-phase rise in venous flow. The maximum flow is reached more rapidly in old marmosets (steps 3 to 8 of the CC) than in young marmosets (steps 3 to 10). In the second périod, arterial flow decreases faster than the venous flow (steps 8 to 16 in old and steps 10 to 16 in young marmosets). The third period, from step 17 onwards, corresponds to the diastole, where the decrease in the venous flow is linearly correlated with a reduction in arterial flow.

The temporal analysis focused on the arrival time, expressed as the percentage of the cardiac cycle, of the peak flow values (represented as point M on the blood flow profile, Fig. 3). The chronological sequence of cardio-synchronised detection of the peak flow in the arteries and sinuses remains consistent in young (YA) and old (OA) marmosets (Fig. 4). The established sequence is as follows for arterial inflows: basilar trunk, left carotid, and right carotid. For venous outflows, the sequence includes the straight sinus followed by the superior sagittal sinus. However, this sequence occurs significantly earlier in the cardiac cycle, regardless of the vessel type, and has a shorter duration in OA than in YA marmosets.

Indeed, the blood flow in BT and the two sinuses peaks significantly earlier in OA than in YA (Fig. 3, lower part). Additionally, it is worth noting that the maximal blood flow is reached 5–6% earlier in the CC for the basilar trunk compared with the carotid arteries (with all velocities being measured on the same brain MRI slice). However, this earlier peak of the basilar trunk is significant only in old marmosets. No significant difference was observed between the two carotid arteries in young or old marmosets (Fig. 3, lower part).

Regarding the venous compartment imaged in a single brain MRI slice for both sinuses, the peak arrival time in the StS significantly precedes that in the SS in the old marmosets (Fig. 3, lower part).

Considering the animals in which arterial and venous flows could be measured (seven marmosets in each group; Supplementary Table S1), the lag between the arterial and venous flow peaks is approximately 50% shorter in OA. This effect is particulary evident for the lag between the carotids and the superior sagittal sinus (SS) (Fig. 3, upper part).

#### 3.2.2 Arterial flows

The total intracerebral arterial blood flow was assessed by combining the flows through the basilar trunk and the two internal carotids throughout a cardiac cycle (Fig. 4A). Arterial blood flow reaches higher values (significant from the fourth to the sixth step of CC) in OA.

Consistent with the chronological sequence (Fig. 3), the time course varies between YA and OA, with the peak of total arterial blood flow occurring earlier in the cardiac cycle for OA (25% CC, step 8) than in that for YA (31% CC, step 10). These results were further supported by the steeper slope of the arterial blood flow time course in OA, significant for BT (23 ± 6 µl/s^2^ in OA *vs.* 16 ± 4 µl/s^2^ in YA, p = 0.007). Slopes were calculated based on the difference between the initial blood flow increase (step 3 of CC = 9.4% CC) and the maximal blood flow value at the corresponding cardiac cycle percentage.

#### 3.2.3 Venous flows

While the dynamics of venous flow are less pronounced than in arteries, the general pattern is similar between YA and OA, with the maximum venous flow value occurring earlier in the CC in OA by approximately 10% CC, both in SS (33.5 ± 5.0% CC in OA *vs.* 44.2 ± 15.6% CC in YA, p = 0.108) and StS (28.6 ± 4.9% CC in OA *vs*. 38.4 ± 9.0% CC in YA, p = 0.052). The flow increases and progresses more smoothly in the young venous system than in the old one (Fig. 4A).

The time course and values of venous blood flow are similar in the SS and StS throughout the cardiac cycle in old marmosets. In the young group, values are higher in StS than in SS (Supplementary Fig. S2).

The relationship between arterial and venous blood flows is illustrated in Figure 4B (see Discussion section).

### 3.3 Cerebral fluid volumes

Thanks to the use of flow data obtained with prospective cardiac gating (triggered by the cardiac signal) and extrapolated over the entire cardiac cycle (CC), we were able to calculate the arterial blood volume entering the brain and the venous blood volume leaving the brain during a single cardiac cycle.

#### 3.3.1 Arterial blood

The right and left carotids contribute similarly to total arterial input regardless of the marmoset age (Table 1). The carotid system (RC + LC) significantly emerges as the primary arterial input in young marmosets. Specifically, the carotids supply 60% of the arterial blood, while 40% comes from the basilar trunk (Table 1) (p = 0.031). In old marmosets, the carotid and the vertebral systems contribute similarly to the arterial blood volume inflow, with 45% from the basilar trunk and 55% from the carotids (p = 0.195). The total arterial blood volume per cardiac cycle increases significantly by 44% in OA compare with that in young marmosets. This increase is primarily due to the vertebral system, which increases by 59%, rather than the carotid system increasing by 35%.

However, the significant difference in the total arterial blood volume per cardiac cycle between YA and OA vanishes when considering perfusion, that is, the blood volume delivered per minute as typically calculated, which is approximately 10 ml/min in both age groups (Table 1). Using the individual anatomical data obtained for each animal on GM and WM volumes, with a brain density of 1.05 g/ml (Stephan et al 1981), we determined the mean cerebral blood flow (CBF, expressed as ml/min/100g of brain tissue). CBF was similar between the age groups (p = 0.535) (Table 1).

**Fig. 5.**
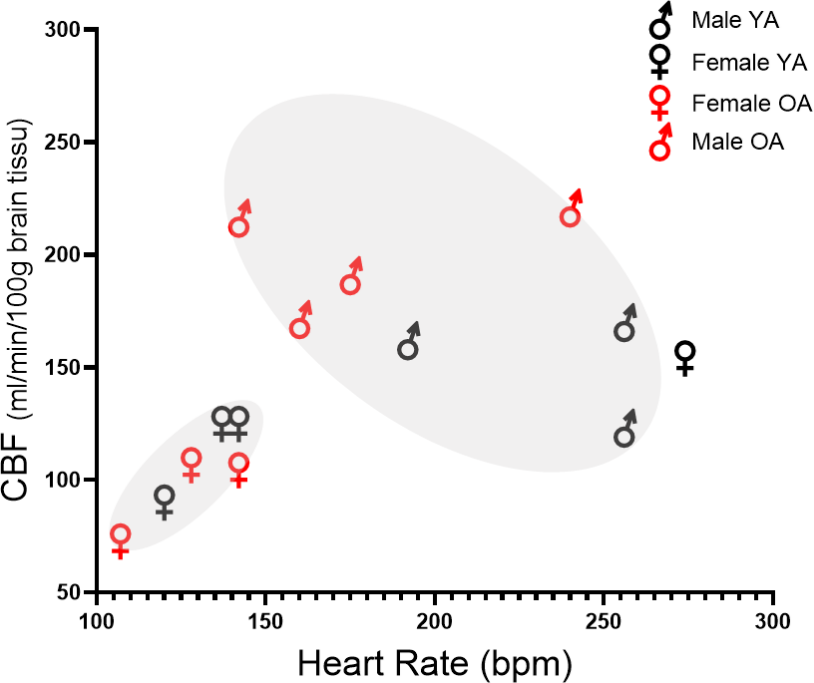
Relationship between heart rate (HR) and cerebral blood flow (CBF) (individual analysis). Data for females and males may segregate based on heart rate (HR) and cerebral blood flow (CBF) values. The data distribution indicates a sex effect on both HR and CBF, while an age effect differentiates young from old males, suggesting an age–sex interaction.

As our age groups were each composed of a similar number of males and females, we explored the potential effect of sex on marmoset CBF during ageing (Supplementary Table S4). We observed that the distribution of individual CBF data as a function of heart rate (Fig. 5) reveals some differences between males and females. The data distribution shows higher CBF values and variability among males than among females (p = 0.014), with a possible age effect in males (p = 0.057) (Supplementary Table S4).

#### 3.3.2 Venous blood

Regarding the blood volume in each sinus and the total venous blood volume (SS+StS) flowing during one cardiac cycle, no significant difference was observed between the two age groups (Table 1). The contributions of the SS and StS are similar between young and old marmosets (Table 1).

The mean value of the total drainage is higher in YA (40.5 ± 11.9 ml/min) than in OA (27.6 ± 14.5 ml/min), p = 0.053 (Table 1).

#### 3.3.3 Inflow/outflow balance

**Fig. 6.**
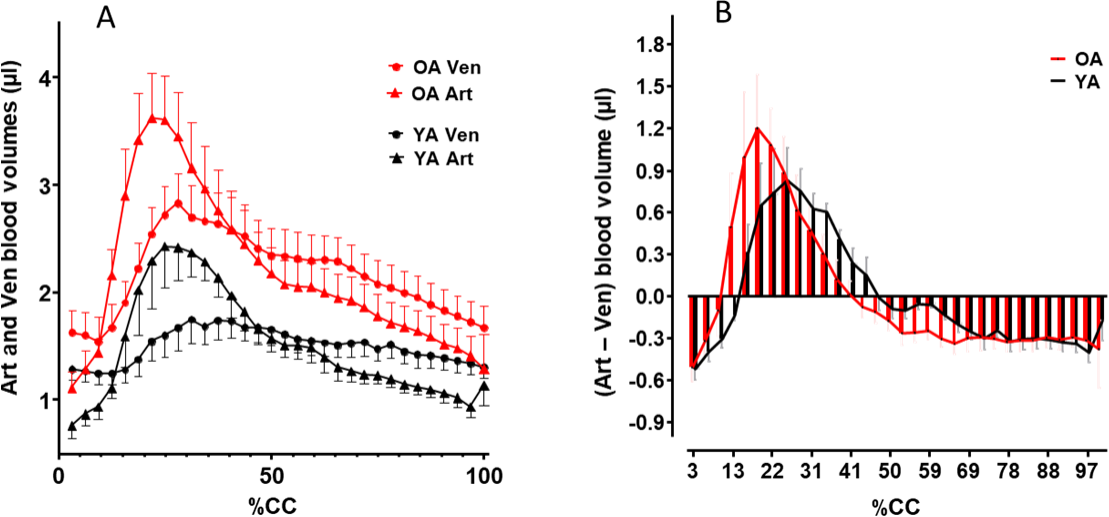
Combined time courses of blood volume in the marmoset brain during the cardiac cycle. (A) Inflow (Art) and outflow (Ven) of blood during the cardiac cycle (CC) (Art = sum of BT, RC, and LC) and venous (Ven = sum of StS and SS) in young (YA) and old (OA) adult marmosets. Data are presented as mean ± SEM (n = 7 for each age group). (B) Evolution of the balance between arterial and venous blood volumes, that is, arterial blood volume minus corrected venous blood volume, along the CC. The excess of blood (Art-Ven > 0) within the brain vascular compartment (positive values) is greater and occurs earlier in OA than in YA marmosets, which is consistent with the earlier peak of arterial blood flow (systolic phase of the CC) observed in aged marmosets.

The sum of the blood volume drained by the two sinuses was lower than the arterial blood volume per CC (Table 1). According to Monro–Kellie doctrine, which describes the principle of homeostatic intracerebral volume regulation, the arterial and venous intracerebral blood volumes must equalise at each cardiac cycle. To analyse the global blood dynamics inside the cerebral tissue, we corrected the venous flow volume by a factor (CF) corresponding to the ratio of arterial to venous blood volume, as described in Baledent et al. (2001). This allowed us to quantitatively assess the respective volumes of the arterial and the venous blood within the brain at each step of the cardiac cycle (Fig. 6A). The time course and amplitude of arterial and venous volumes differ between YA and OA, as expected from the brain inflow and outflow analysis.

We quantitatively assessed the temporal progression of the balance between arterial and venous blood volumes by calculating the difference between the two at each CC step. This analysis indicates that intracerebral blood volume is not balanced along the CC, as indicated by the non-zero values of arterial blood volume minus venous blood volume (Art-Ven). Consequently, the blood compartment experiences alternating phases of blood deficit (negative values) and surplus (positive values) during the CC (Fig. 6B). The maximum blood excess (overload) occurs earlier and reaches higher values in OA than in YA during the CC.

The oscillating volume of blood (Art-Ven) is 5.8 ± 2.7 µL in YA and 7.4 ± 2.3 µL in OA. This volume would correspond to the CSF volume displaced from the intracranial to the extracranial compartment during the CC to maintain the physiological intracranial pressure in accordance with the Monro–Kellie doctrine. On average, this volume represents 0.72 ± 0.03% and 0.77 ± 0.05% of the intracranial CSF volume in YA and OA, respectively, with no significant difference between the two age groups.

## 4 Discussion

### 4.1 Validation

PC-MRI enables non-invasive and reliable measurements of blood flow velocity throughout the cardiac cycle in humans (Alperin et al., 1996; Balédent et al., 2001; Enzmann et al., 1994). The present work reports on the first PC MRI analyses using prospective cardiac gating in marmosets, aiming to capture the temporally coordinated arterial and venous blood flows in healthy monkeys.

Our baseline data align with previously published studies on cerebral anatomy (C. Liu et al., 2021; Sawiak et al., 2018; Seki et al., 2017). Seki et al. (2017) reported that the parenchyma (GM + WM) occupies 6.73 cm^3^, a value very close to our findings (6.94 ± 0.77 cm^3^). Our data are also in line with the literature, which shows that brain, GM and WM volumes are not different between males and females (C. Liu et al., 2021; Seki et al., 2017).

Heart rate averages 230 bpm in marmoset but substantially increases under restraint (Mietsch & Einspanier, 2015; Schnell & Wood, 1993). Comparatively, our recorded data were lower (~190 in young adults and 150 bpm in older adults, Table 1), consistent with the cardiac rate recorded in anaesthetised marmosets (Klösener et al., 2023; Moussavi et al., 2020). No significant age-related difference was reported either (Moussavi et al., 2020).

We carefully optimise the experimental conditions to ensure accurate blood velocity in marmoset intracranial vessels and sinuses (see Material and Methods). The mean peak velocity in the arterial vessels was approximately 15 cm/s in our experiments. In comparison, the velocity peak obtained with PC-MRI for the carotid in humans is ~35 cm/s (estimated from Balédent et al. (2001)). Anaesthesia may affect blood velocity; however, in isoflurane-anaesthetised marmosets, vessel dilation associated with blood velocity decrease prominently occurs in small-diameter cortical vessels (Santisakultarm et al., 2016).

Comparing reported blood flow values across studies is challenging due to methodological differences. Generally, authors do not specify the method they used. Indeed, the blood velocity given at each CC step can be defined as the value identified in the voxel with the maximal intensity among the voxels, or as the mean of the velocities among the voxels of the artery section (Seitz et al., 2001). In our experiments, blood velocity could reach tens of cm/s in at least one voxel per vessel section, a value corresponding to arterial blood velocity values reported in humans.

Finally, we could compare our marmoset experimental data with blood flow simulations calculated in different primate species (Seymour et al., 2015). Our findings indicate an average brain perfusion of 10 ml/min and a mean brain volume of 8 ml in marmosets. These values are close to Seymour’s scaling prediction (Seymour et al., 2015) in primates (perfusion = brain volume^0.95^), leading to a theoretical perfusion of 7.2 ml/min for an 8 ml marmoset brain. However, Seymour’s model considers only the carotid inlet, as noted by Boyer and Harrington (2018). Considering that the carotid system in marmosets contributes to 60% of the total arterial brain blood flow (cf. results), theoretical perfusion should be closer to a dozen ml/min.

Futhurmore, we could conclude that in marmosets, the ratio of arterial inflow (as measured in our experiments) to the mean cardiac stroke volume in adult marmosets (measured by MRI; Klösener et al., 2023; Moussavi et al., 2020) corresponds to 9–14% of total cardiac output. Despite experimental differences between marmoset and human (prone/supine position (Alperin et al., 2005), anaesthetised/vigil condition (Mckinney et al., 1993)), our results appear physiologically coherent and consistent given the ~15% of cardiac flow output to the brain in humans (Williams & Leggett, 1989).

### 4.2 Haemodynamics in aged marmoset brain

Our sample showed no phenotypical differences between males and females or between young and old marmosets in terms of body weight and brain size. However, measurements of vascular functionality revealed statistically significant differences between young and old adult marmosets, occuring at the cardiac cycle scale.

#### 4.2.1 Age effect

The sequence of blood inflow and outflow peaks in the intracerebral compartment is similar between the two age groups but starts earlier in the cardiac cycle and takes place on a shorter time window in the older marmosets. Moreover, we observed a dissociation among the arterial inputs in old animals: the flow peak of the vertebral system precedes that of the carotid system, which occurs simultaneously in younger marmosets. Similarly, the venous peak in the straight sinus precedes this of the sagittal sinus in older marmosets. In humans, differences in intracranial arterial and venous flow waveform timing as an effect of age in healthy individuals are not commonly described (Enzmann et al., 1994; Schmid Daners et al., 2012).

We calculated the pulsatility index PI (PI = (max flow – min flow)/mean flow) (Gosling & King, 1974) and the resistive index (Pourcelot index, RI = (max flow-min flow)/max flow) typically used to assess the vascular resistance in pulsatile systems. PI and RI are higher, although not significantly, in old marmosets in all arterial vessels and sinuses (e.g., PI values for OA *vs.* YA: 1.51 ± 0.51 *vs.* 1.48 ± 0.57 for BT, 1.37 ± 0.65 *vs.* 1.03 ± 0.42 for RC, 1.65 ± 1.08 *vs.* 1.29 ± 0.34 for LC).

These observations indicate an alteration in the cardiovascular system (stiffening of input vessels) and a modification in the compliance of the vascular tissue and parenchyma within the brain. Modifications in the arterial wall structure have been well described in the ageing human carotids (Pewowaruk et al., 2022). Alterations in the mechanical properties of the brain tissue (Parker et al., 2024) and vascular drainage are less well documented (Kalaria, 1996), although they may control the level of pressure within the brain parenchyma and the intracerebral vascular bed (Hakim et al., 1976). Very few biochemical and structural alterations linked to the intracerebral venous system have been reported in marmosets older than 6 years (Brown et al., 2002; Sobin et al., 1992) and in humans (Shaaban et al., 2017).

Due to technical limitations, we were unable to measure blood pressure (BP) during the MRI experiments, which would have provided useful information on the biomechanical vessel properties for both age groups. Developing a non-invasive, MRI-compatible device for BP measurement in our marmoset sample would be required for future studies.

#### 4.2.2 Sex effect

Brain volumes, as well as white matter or grey matter volumes, do not differ significantly between male and female marmosets, while HR is higher in males than in females. Although examined in small sample sizes, we observed a sex effect on CBF, which may interact with an age effect (Fig. 5; Supplementary Table S4).

Such an effect cannot be explained by a different age sampling between the sexes, the age range being narrow and similar in the sex/age groups (Supplementary Table S1). Nevertheless, age-associated differences appear prominent in males (e.g., lower heart rate in old marmosets, increased arterial blood flow in older males; Supplementary Table S4). These observations suggest that sex may impact brain haemodynamics ageing and should be considered when studying brain fluid characteristics. Further explorations using larger samples are required to investigate the effect of age, particularly on cardiac volume, ejection fraction, brain cardiac output, and processes of cardiovascular regulation in male and female marmosets. Published data in humans do not agree on the effect of sex or the interaction between sex and age on brain fluids (Alisch et al., 2021; Owashi et al., 2023; Sartoretti et al., 2019; Schmid Daners et al., 2012).

### 4.3 Comparative arterio-venous coupling in young and old marmosets

To summarise our findings on the brain arterio-venous coupling at the cardiac cycle scale in young and old marmosets (Fig. 4), we analysed the temporal evolution of arterial perfusion and venous drainage (quantitative values in µl/min) over the cardiac cycle (normalised in % CC) in the two age groups. The complex relationship between input flow and output flow during the cardiac cycle (Fig. 4A), particularly with the phase difference between input and output, results in a characteristic hysteresis effect (Fig. 4B). Because vessels and brain tissue possess specific viscoelastic properties, mechanical energy is stored during systole as they deform under pressure. This stored energy is gradually released during diastole; however, due to the viscous nature of the vessels and brain tissue, this release is delayed, creating a phase lag that explains the nonlinear relationship between arterial and venous flow throughout the cardiac cycle. This relationship comprises three successive periods: the first two periods correspond to systole (steps 3–16), and the third period to diastole (steps 17–32). We propose that the surface area in the hysteresis cycle, which reflects the impact of the cranial system’s fluid and tissue biomechanical constraints on brain blood behaviour, might correspond to the transfer function of blood flow to CSF flow, according to Monro–Kellie doctrin. The old/young ratio value of surface area (1.25) is close to that of the difference between arterial and venous volumes (Art-Ven) (1.27). Interestingly, we have assumed that this (Art-Ven) volume is correlated with the CSF volume buffering the variation in intracerebral volume during the cardiac cycle (Capel et al., 2023). This volume variation corresponds to less than 1% of the cranial CSF volume, less than 0.1% of the cerebral volume, and ~11–12% of the arterial blood volume. An (Art-Ven) volume of 0.82 ml/CC has been found in aged patients (Capel et al., 2023), leading to similar values when considering a brain volume of 1100 ml and an arterial blood volume of 11 ml per CC in humans.

Age discriminates between the two hysteresis cycles: they share similar orientation and shape but differ in a translation along the y-axis (venous flow). Arterial perfusion spans the same range of values in young (92–236 ml/min) and old marmosets, although the range is larger in old marmosets (113–298 ml/min). Strikingly, the range of venous drainage values is shifted towards lower values in the old marmoset group (20–35 ml/min *vs.* 34–46 ml/min in the young group). Indeed, the difference between the mean arterial and mean venous flows over a cardiac cycle is greater in old (156.50 ± 67.76 ml/min) than in young (110.34 ± 34.30 ml/min) marmosets, though this difference is not significant. The quantitative difference between arterial and venous flows is a common observation in similar studies performed in humans and is explained by an underestimation of the venous flow due to peripheral venous drainage, which is not measured in the experiments (see the Limitations section below). The authors adress this issue by applying a correction factor (CF = arterial flow/venous flow), for which age differences have not been reported in humans (Bateman et al., 2008; Lokossou et al., 2020). Our findings indicate higher, though not significantly different, CF values in old (7.40 ± 2.26) than in young (5.85 ± 2.69) marmosets, suggesting a more substantial peripheral venous contribution in the older group. Therefore, the drainage of cerebral blood does not depend solely on arterial perfusion but also on age-dependent factors linked to the mechanical properties of venous vessels and sinuses.

### 4.4 Limitations

The number of marmosets included in the present study is a concern for our analysis. However, the age range within each age group is narrow, and both males and females are represented. Our cross-sectional study is a preliminary approach to further exploring the ageing process through a longitudinal study that assesses brain haemodynamics and structure in the same individuals throughout their lifetime.

Comparisons between marmoset and human data suffer from some differences in the experimental design. Marmosets are anaesthetised and positioned prone during imaging sessions, whereas human subjects are awake and lie supine. Both anaesthesia and body/head positions can impact blood flow, for example, through vessel diameter, vessel stretch, pressure, and gravity effects. General observations suggest that isoflurane induces vasodilation and, according to some authors, increases blood velocity, enhancing cerebral perfusion (Cao et al., 2017; Santisakultarm et al., 2016; Sullender et al 2022). These changes can also alter fMRI signals (Hori et al., 2020). A key question for our study is whether anaesthesia exerts age-dependent effects on the cerebrovascular system. In awake young individuals, including adolescent humans, baseline CBF is higher than in older individuals (Biagi et al., 2007; Satterthwaite et al., 2014). A similar age-related pattern in CBF has been observed in anaesthetised mice (Munting et al., 2021). To our knowledge, no study has specifically examined the age-dependent effects of isoflurane. While a confounding effect of isoflurane on age-related vascular changes cannot be ruled out, its impact on CBF differences across age groups is likely moderate. Nonetheless, MRI studies with awake marmosets restrained in a sphynx position can be further explored, although such methods are typically employed for fMRI explorations.

Furthermore, intrinsic differences exist in the biology of the two primate species (e.g., the absence of menopause in female marmosets) and in brain anatomy (the marmoset‘s lissencephalic brain). Although these biological and anatomical differences cannot be eliminated, they can be informative for data interpretation.

Additonally, literature data are scarce in assessing a strict similarity in the intracranial vascular organisation between humans and marmosets. Past studies (Beattie, 1927; Freret et al., 2008; Puentes et al., 2015) and our data describe an organisation of the arterial inputs and venous sinuses in marmosets that is similar to that in humans.

Importantly, we were able to record blood velocity in all the arterial inputs to the brain on the same slice, but we could not measure it in all the intracerebral venous outputs. While we imaged venous blood flow in the superior sagittal and straight sinuses on the same brain slice, blood flow in peripheral veins could not be captured. These peripheral veins (the Trolard and de Labbe anastomotic veins) and the petrosal sinus drain into the transverse sinus downstream of the sagittal sinus. These pathways may represent a significant fraction of the brain drainage system. Consequently, our analysis underestimated the intracerebral venous output. We corrected this by equating the arterial input and venous output values. Correction factors (arterial flow/venous flow) are commonly calculated in human subjects, where discrepancies between total arterial and venous intracerebral blood flows are observed in studies similar to ours (Balédent et al., 2001; Bateman, 2000; Enzmann et al., 1993; Lokossou et al., 2020).

## 5 Conclusion

This study highlights two key points. First, it demonstrates the feasibility of non-invasively tracking cerebral fluid dynamics in non-human primates across the cardiac cycle and potentially over a lifetime through longitudinal studies. Second, analysing arterial and venous flows provides insight into the dynamic coupling between blood compartments and changes at the cardio-cerebral and cerebrovascular levels. The findings reveal age-related effects, with a possible sex influence warranting further investigation. During normal ageing, we observed temporal and quantitative changes in arterial blood flow occurring early in systole, accompanied by modified venous drainage. These haemodynamic changes may, in turn, impact CSF dynamics. Studying these age-related modifications, which reflect changes in cranio-spinal compliance, is essential for characterising pathological brain ageing (Bateman, 2000; Owashi et al., 2023; Stoquart-ElSankari et al., 2007). This work lays the foundation for a similar non-invasive approach to exploring blood and CSF flow interactions in this preclinical model.

## Supplementary Material

Supplementary Figure S1

Supplementary Figure S2

Supplementary Table S1

Supplementary Table S2

Supplementary Table S3

Supplementary Table S4

## Data Availability

Data generated and analysed during the study are included in the Supplementary Information (Supplementary Table S3).
