# Supplementary material for: Arterial and venous flow dynamics are modified by age in the non-human primate
Source: Imaging Neurosci (Camb). 2025 Jul 7;3:IMAG.a.66. doi: 10.1162/IMAG.a.66 (PMC12330867; doi:10.1162/IMAG.a.66)
Supplement: Supplementary Figure S1 [file IMAG.a.66_supp_FigS1.pdf]

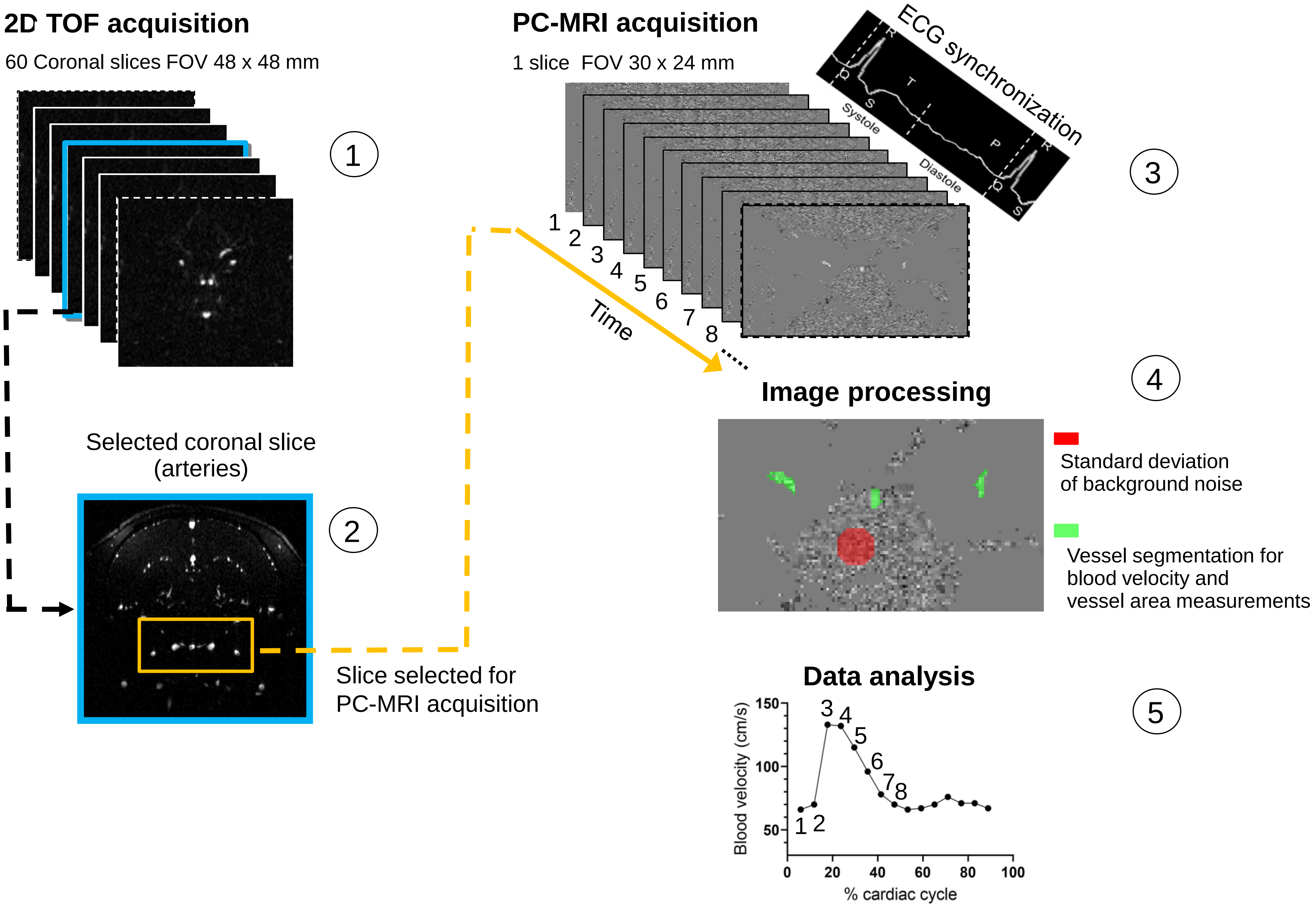

Fig S1: Image acquisition and data analysis pipeline
