# Supplementary material for: Arterial and venous flow dynamics are modified by age in the non-human primate
Source: Imaging Neurosci (Camb). 2025 Jul 7;3:IMAG.a.66. doi: 10.1162/IMAG.a.66 (PMC12330867; doi:10.1162/IMAG.a.66)

Figure S2: Time course of blood flows in straight and superior sagittal sinuses during the whole cardiac cycle in young and old adult marmosets.

StS: straight sinus, SS: superior sagittal sinus, CC: cardiac cycle
YA: young marmosets, OA : old marmosets.
Data are presented as mean ± SEM (n=7 for each age group)

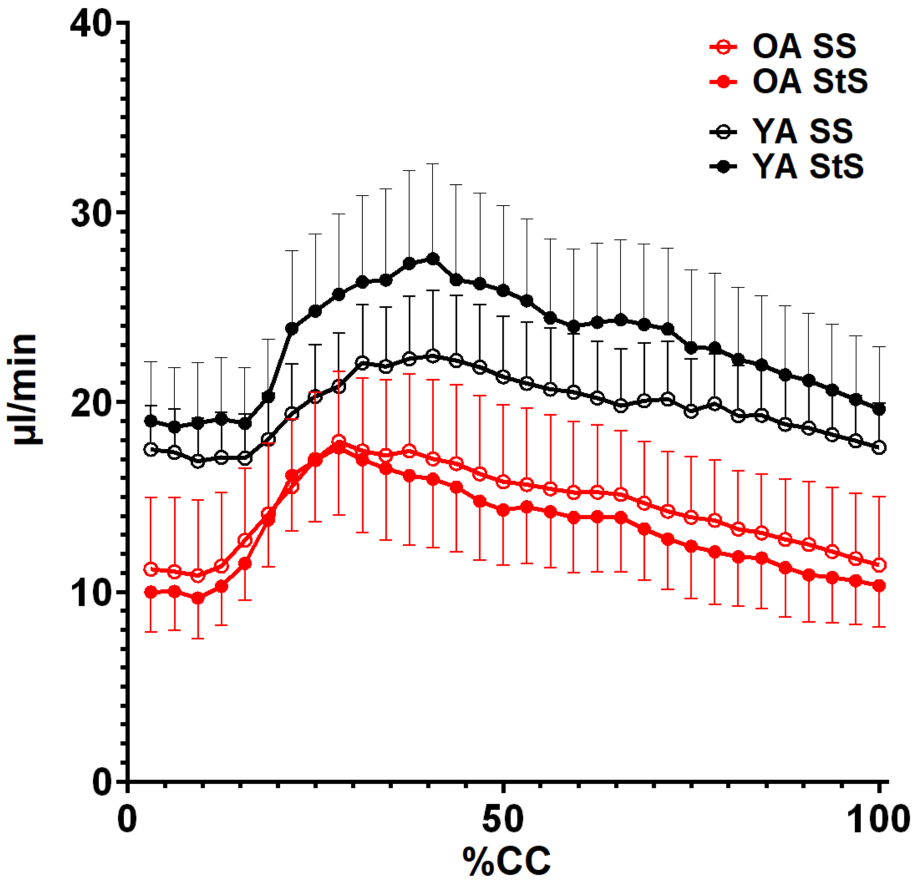

Supplement: Supplementary Figure S2 [file IMAG.a.66_supp_FigS2.pdf]
