# Supplementary material for: Arterial and venous flow dynamics are modified by age in the non-human primate
Source: Imaging Neurosci (Camb). 2025 Jul 7;3:IMAG.a.66. doi: 10.1162/IMAG.a.66 (PMC12330867; doi:10.1162/IMAG.a.66)
Supplement: Supplementary Table S1 [file IMAG.a.66_supp_TableS1.pdf]

Table S1 : Summary of marmosets' biological characteristics and experimental acquisitions. f=female, m=male, bpm = beats per minute, CC = cardiac cycle

T2-W = T2 weighted image, PC = phase contrast, TOF = Time Of Flight
BT = basilar trunk artery, LC = left carotid, RC = right carotid, SS = superior sagittal sinus, StS = straight sinus.

Adult marmosets	Sex	Weight g	Age years (days)	Brain anatomy (T2-W)	Angiography (TOF)	Hemodynamics (PC-MRI)	Heart rate in bpm (CC duration in ms)		PC-MRI acquisition duration in % CC length	
							PC artery acquisition	PC sinus acquisition	PC artery acquisition	PC sinus acquisition
**Young - YA**										
Mar 001	m	330	5.0 (1820)	x	x	RC,LC,BT,SS, StS	256 (234)	240 (250)	76.80	72.00
Mar 003	m	390	4.0 (1465)	x	x	RC,LC,BT,SS, StS	256 (243)	226 (265)	83.20	73.41
Mar 006	m	330	4.0 (1468)	x	x	RC,LC,BT,SS, StS	192 (313)	202 (297)	72.00	75.79
Mar 007	m	335	3.6 (1318)	x	x	BT, SS, StS	175 (343)	192 (313)	82.90	86.40
Mar 009	m	400	4.3 (1553)	x	x	no data	no data	no data	no data	no data
Mar 012	f	330	4.8 (1750)	x	x	RC,LC,BT,SS, StS	274 (219)	256 (243)	82.30	76.80
Mar 013	m	430	3.9 (1440)	x	x	RC, LC, SS, StS	154 (390)	137 (438)	76.80	68.57
Mar 016	f	360	3.2 (1176)	x	x	RC,LC,BT,SS, StS	137 (438)	124 (484)	85.70	83.61
Mar 018	f	430	3.4 (1238)	x	x	RC,LC,BT,SS, StS	120 (500)	132 (455)	75.00	82.76
Mar 020	f	400	3.2 (1157)	x	x	RC,LC,BT,SS, StS	142 (423)	132 (455)	88.90	82.76
**Old - OA**										
Mar 002	m	330	9.0 (3283)	x	x	RC,LC,BT,SS, StS	142 (423)	132 (455)	53.33	49.66
Mar 004	m	450	9.0 (3273)	x	x	RC,LC,BT,SS, StS	240 (250)	240 (250)	90.00	90.00
Mar 005	f	350	8.8 (3213)	x	x	RC,LC,BT,SS, StS	107 (561)	124 (484)	40.00	46.45
Mar 008	f	430	9.0 (3277)	x	x	no data	no data	no data	no data	no data
Mar 011	f	400	8.9 (3265)	x	x	RC,LC,BT,SS, StS	142 (423)	128 469)	78.20	70.40
Mar 014	f	400	9.2 (3371)	x	x	RC,LC,BT,SS, StS	128 (469)	116 (517)	76.80	81.45
Mar 015	m	398	8.4 (3071)	x	x	RC,LC,BT,SS, StS	175 (343)	142 (423)	78.50	64.00
Mar 017	m	360	8.4 (3052)	x	x	RC, LC, BT	120 (500)	no data	66.00	no data
Mar 019	m	400	8.4 (3053)	x	x	RC,LC,BT,SS, StS	160 (375)	142 (423)	80.00	81.78
