# Supplementary material for: Arterial and venous flow dynamics are modified by age in the non-human primate
Source: Imaging Neurosci (Camb). 2025 Jul 7;3:IMAG.a.66. doi: 10.1162/IMAG.a.66 (PMC12330867; doi:10.1162/IMAG.a.66)
Supplement: Supplementary Table S2 [file IMAG.a.66_supp_TableS2.pdf]

Table S2 : Repeatability metrics for cross-sectional area, velocity and blood flow. Coefficients of variation (CV) and intra-class correlation coefficients (ICC) computed from two independent analyses on n = 4 marmosets (2 young adults, 2 old adults). For the internal carotid arteries, values are averaged over left and right carotids (RC + LC).

Vessel	Cross-Sectional Area CV (%)	Velocity CV (%)	Blood Flow ICC
**Basilar artery (BT)**	8.2	7.5	0.92
**Internal carotid arteries (RC + LC)**	9.6	8.8	0.90
**Superior sagittal sinus (SS)**	12.5	11.0	0.85
