# Supplementary material for: Arterial and venous flow dynamics are modified by age in the non-human primate
Source: Imaging Neurosci (Camb). 2025 Jul 7;3:IMAG.a.66. doi: 10.1162/IMAG.a.66 (PMC12330867; doi:10.1162/IMAG.a.66)
Supplement: Supplementary Table S4 [file IMAG.a.66_supp_TableS4.pdf]

Table S4 : Cerebral blood supply in male or female marmosets. Data are presented as mean ± standard deviation and compared with Mann-Whitney Test.

	**Males (n)**	**Females (n)**	p
**Heart Rate (bpm)**			
All	203 ± 47 (7)	150 ± 56 (7)	0.034
Young	234 ± 37 (3)	168 ± 71 (4)	0.284
Old	179 ± 43 (4)	126 ± 18 (3)	0.050
**Arterial blood volume (µl/CC)**			
Young	45.0 ± 12.2 (3)	49.8 ± 11.1 (4)	0.857
Old<br>(% increase old *vs* young)	81.1 ± 14.3 (4)<br>(+ 80%)	53.4 ± 7.0 (3)<br>(+7%)	0.057
Young vs Old	*p = 0.057*	*p = 0.857*	
**Perfusion (ml/min)**			
All	12.5 ± 2.7 (7)	7.5 ± 2.2 (7)	0.004
Young	10.3 ± 1.2 (3)	8.1 ± 2.7 (4)	0.400
Old	14.2 ± 2.1 (4)	6.7 ± 1.3 (3)	0.057
Young vs Old	*p = 0.057*	*p = 0.629*	
**CBF** (ml/min/100g tissue)			
All	168.5 ± 36.3 (7)	106.7 ± 24.8 (7)	0.004
Young	135.6 ± 20.9 (3)	119.5 ± 20.5 (4)	0.400
Old	193.2 ± 21.0 (4)	89.6 ± 21.0 (3)	0.057
Young vs Old	*p = 0.057*	*p = 0.288*	
